# Hepatoprotective potential of N-acetyl cysteine in rats with phenytoin induced liver injury

**DOI:** 10.12688/f1000research.163160.1

**Published:** 2025-06-17

**Authors:** Noor D. Aziz, Deema Diyaa Azeez, Amal Umran Mosa, Zahraa Abed Al-kareem, Sahar A. Majeed, Fadhaa Abdulameer Ghafil

**Affiliations:** 1 Department of Clinical Pharmacy, College of Pharmacy, University of Kerbala, Kerbala, Kerbala, Iraq; 2 Department of Pharmaceutics, College of pharmacy, University of Kerbala, Kerbala, Iraq; 3 Department of pharmacology and toxicology,, College of pharmacy, University of Kerbala, Kerbala, Iraq; 4 Department of pharmacology and therapeutics, Faculty of medicine, Kufa University, Kufa, Iraq

**Keywords:** Hepatoprotective, N-acetyl cysteine, phenytoin, liver injury

## Abstract

**Background:**

Phenytoin is an anticonvulsant medication that is effective in treating various seizure disorders. It is mostly metabolized by the liver, which increases the risk of PHT-induced hepatotoxicity.

**Aims:**

This study aimed to assess the effectiveness of N-acetylcysteine (NAC) in protecting the liver from phenytoin-induced hepatotoxicity in rats.

**Materials and Methods:**

Four sets of five rats male Wistar albino rats (Rattus norvegicus) used for this study was based on their availability, well-established physiology, and long history of use in pharmacological and toxicological studies each were used for analysis. Each of the four groups received different treatments: the control group received normal saline, one group received 200 mg/kg/day of NAC, another group received 5 mg/kg/day of phenytoin, and the fourth group received 200 mg/kg/day of both phenytoin and NAC. The treatments were administered orally by gavage for 45 days. Biochemical indicators (aspartate aminotransferase (AST), alanine aminotransferase (ALT), alkaline phosphatase (ALP), and total serum bilirubin (TSB)) were measured in serum after the animals were anaesthetized and the experiment ended. Histological analysis was performed on liver specimens.

**Results:**

Our investigation showed that phenytoin significantly elevated liver enzymes and total serum bilirubin compared to the control and NAC groups. The concurrent administration of NAC and phenytoin led to a notable reduction in these biomarkers, excluding ALP levels. Moreover, the group that received NAC alone did not exhibit a significant increase in the levels of these biomarkers compared with the control group. The histopathological results were in agreement with the biochemical tests.

**Conclusion:**

This study concluded that Concomitant administration of NAC and phenytoin lowered the risk of phenytoin-induced hepatotoxicity. Moreover, this study confirmed that NAC is relatively safe when administered for a relatively prolonged period.

## Introduction

Phenytoin (PHT) is a medication used to treat neurological and psychiatric conditions, as well as epilepsy (antiepileptic). ^1^ PHT metabolites are associated with liver damage, including cholestatic hepatitis, cytotoxic hepatitis, or mixed reaction. ^2, 3^ The exact mechanism of PHT-induced hepatotoxicity remains unclear. ^4^ One of the proposed mechanisms is that PHT may cause significant liberation of reactive oxygen species in hepatic mitochondria, resulting in mitochondrial malfunction. ^3, 5^ Another proposed mechanism is thought to be related to the activation of inflammatory pathways with the overproduction of pro-inflammatory cytokines. ^6, 7^ N-acetyl cysteine (NAC) has been used in therapeutic contexts for numerous years. It has been employed in the management of several conditions, including paracetamol overdose, adriamycin-induced cardiotoxicity, ischemia-reperfusion injury, heavy metal poisoning, and idiopathic pulmonary fibrosis. ^8, 9^ The presence of a principal function as an antioxidant, its free thiol group, enables it to interact with reactive oxygen and nitrogen species. ^10, 11^ NAC possesses anti-inflammatory properties by inhibiting the nuclear factor kappa-light-chain-enhancer of activated B cells (NF-κB), a key player in the inflammatory cascade and immune response to oxidative stress. NAC inhibits the translocation of the NF-κB transcription factor and nuclear activation, which are essential for controlling the expression of genes that promote inflammation. ^12, 13^ Research has demonstrated that NAC inhibits the secretion of inflammatory cytokines, such as TNF-α, IL-1β, and IL-6, in macrophages that have been activated by lipopolysaccharide. ^14^ That being said, a number of studies were carried out to investigate the potential hepatoprotective effects of NAC against various hepatotoxic substances, including carbamazepine, ^15^ Adriamycin, ^16^ anti-tuberculosis drugs, ^17^ and others. This study aimed to evaluate the possible protective effects of NAC against PTH-induced hepatotoxicity.

## Ethical approval

This study was conducted with the approval of the ethical committee of the College of Pharmacy, University of Kerbala on February 6, 2024 (Ref: 2024An.8).

## Methods

Twenty male Wistar albino rats, weighing 120–170 g, were used in this study. The animals were kept in the animal house at the College of Pharmacy, University of Kerbala, where they had unlimited access to food and drink, 12-hour light/dark cycle. The animals were acclimated for seven days prior to the experiment, were in good health, and were free of specific pathogens. All animal procedures in this study were conducted in accordance with institutional and international guidelines for the ethical treatment of animals. They were divided randomly into four groups, each of which contained five animals, as follows:

1. The control group administered normal saline only
2. NAC group: NAC (200 mg/kg/day) administered daily for 45 days
3. Phenytoin-treated group: received phenytoin at a dose of 5 mg/kg/day orally for 45 days
4. Phenytoin + NAC group: received phenytoin as in the third group plus NAC (at a dose of 200 mg/kg/day) 1 h before phenytoin daily for 45 days

The animals were maintained for 24 h following their last dose and then anesthetized with xylazine and ketamine (75 mg/kg). After the rats were anesthetized, a 5 cc syringe was used to draw blood from their left ventricle. After centrifuging the serum with an Eppendorf apparatus, it was chilled to -20°C for storage. ^18^ For histological investigation, liver tissues were removed, washed with cold phosphate buffer (pH 7.4), weighed, and stored in 10% formalin for later analysis. ^19^ Biochemical markers, including enzymes that measure total serum bilirubin TSB, ALT, AST, and alkaline phosphatase activity, were assessed via the colorimetric method, utilizing cell biolabs assay kits in compliance with the manufacturer’s protocols.

Throughout the study, no unanticipated or anticipated negative events were noted. All efforts were made to minimize animal suffering, including the use of appropriate anesthesia and analgesia, continuous health monitoring by trained personnel, and the implementation of humane endpoints when necessary. These measures were undertaken to ensure the ethical treatment of animals and the reliability of scientific outcome according to ARRIVE reporting guidelines. ^20^

### Histopathological analysis

The liver was removed and preserved in 10% formalin solution for histopathological examination. Afterwards, the livers were dehydrated using alcohol at escalating concentrations (80-100%, v/v) and then placed in paraffin blocks. ^21^ These blocks were then cut into sections of 4-6 μm using a Rotary Microtome. The organ slices were stained with hematoxylin and eosin (H&E) to assess the tissue morphology using light microscopy. The evaluation of tissue morphology was performed blindly by an expert histopathologist.

### Statistical analysis

SPSS version ^20^ to report the results as mean ± SD, and one-way analysis of variance (ANOVA) https://www.ibm.com/products/spss-statistics was used to examine the statistical significance of differences between the experimental groups. P of 0.05 or less indicates that there were significant differences. Blinding considered (during the allocation, the conduct of the experiment, the outcome assessment, and the data analysis).

## Results

### Effects of phenytoin and N-acetyl cysteine on the serum level of biochemical markers

**Figure 1.**
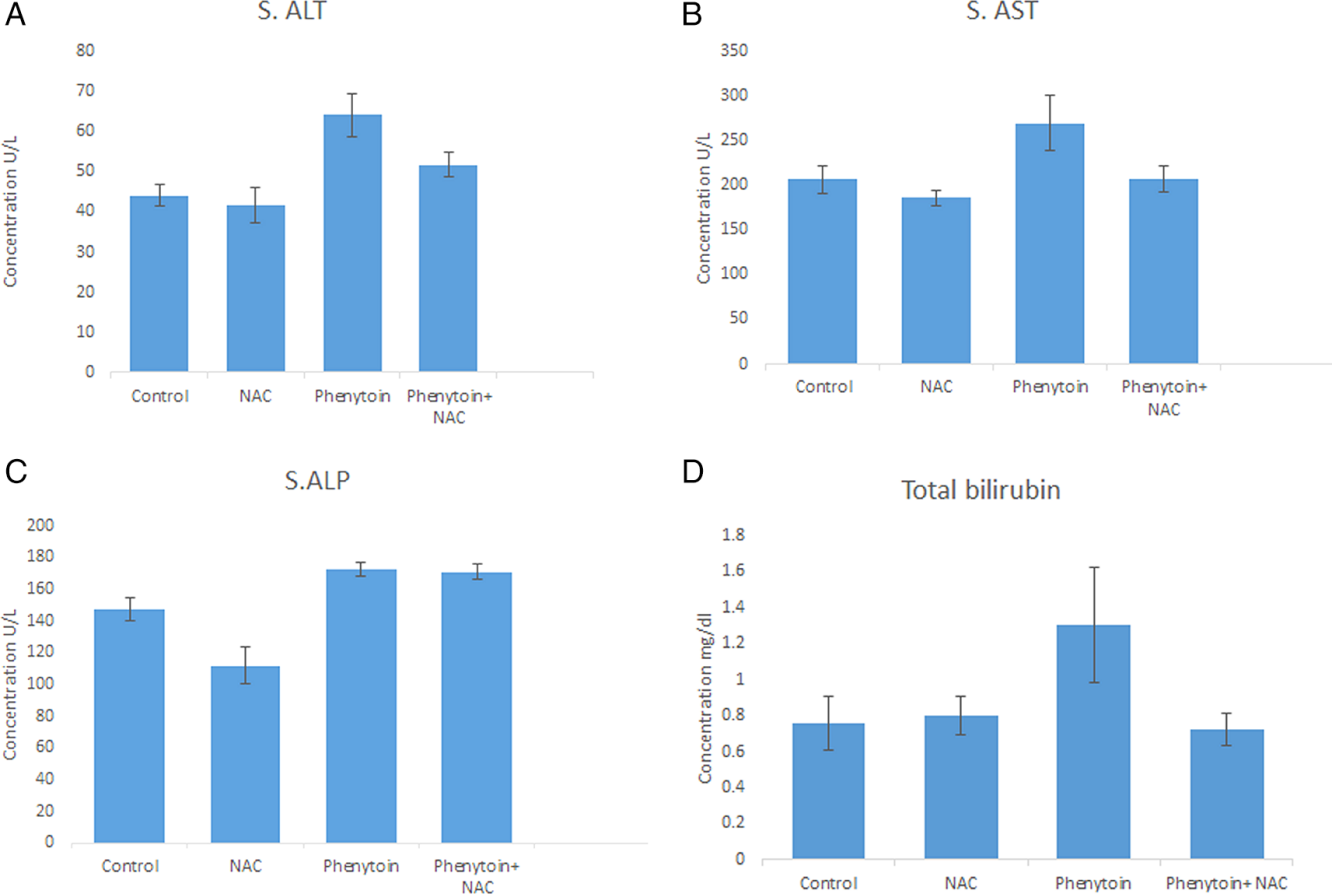
The serum parameters of all groups of rats as follows: A: alanine aminotransferase (ALT), B: aspartate aminotransferase (AST), C: alkaline phosphatase (ALP), D: total bilirubin. The results indicate the mean value ± standard deviation of the mean. a: significant from control, b: significant from NAC, c: significant from phenytoin, d: significant from Phenytoin+ NAC.

The findings revealed a marked increase p < 0.05 in plasma levels of alanine aminotransferase (ALT), aspartate aminotransferase (AST), total serum bilirubin (TSB), and alkaline phosphatase (ALP) in the phenytoin-treated group when compared with the other groups. In addition, there was a significant decrease in the serum levels of ALT, AST, and TSB (except for ALP) in the phenytoin+ NAC group when compared with the phenytoin-treated group. Moreover, in the NAC only treated group, the levels of liver enzymes were low compared to the control group, but this reduction was significant only for ALP level, as shown in Table 1, Figure 1: (A-D).

**Table 1.** Effects of different treatment modalities on biochemical markers.

Parameters\Groups	ALT (U/L) Mean ± SD	AST (U/L) Mean ± SD	ALP (U/L) Mean ± SD	Total Bilirubin (mg/dl) Mean ± SD
Control	44.14 ± 2.79 ^c^ ^,^ ^d^	206.43 ± 15.64 ^c^	147.13 ± 7.59 ^b^ ^,^ ^c^ ^,^ ^d^	0.76 ± 0.15 ^c^
NAC	41.56 ± 4.44 ^c^ ^,^ ^d^	185.72 ± 8.84 ^c^	111.87 ± 11.93 ^a^ ^,^ ^c^ ^,^ ^d^	0.80 ± 0.11 ^c^
Phenytoin	64.16 ± 5.24 ^a^ ^,^ ^b^ ^,^ ^d^	269.64 ± 31.29 ^a^ ^,^ ^b^ ^,^ ^d^	172.16 ± 4.19 ^a^ ^,^ ^b^	1.30 ± 0.32 ^a^ ^,^ ^b^ ^,^ ^d^
Phenytoin+ NAC	51.74 ± 3.04 ^a^ ^,^ ^b^ ^,^ ^c^	207.04 ± 13.97 ^c^	170.82 ± 4.86 ^a^ ^,^ ^b^	0.72 ± 0.09 ^c^

^a^ Significant from control,

^b^ Significant from NAC,

^c^ Significant from phenytoin, and

^d^ Significant from Phenytoin+ NAC.

### Results of histopathology

**Figure 2.**
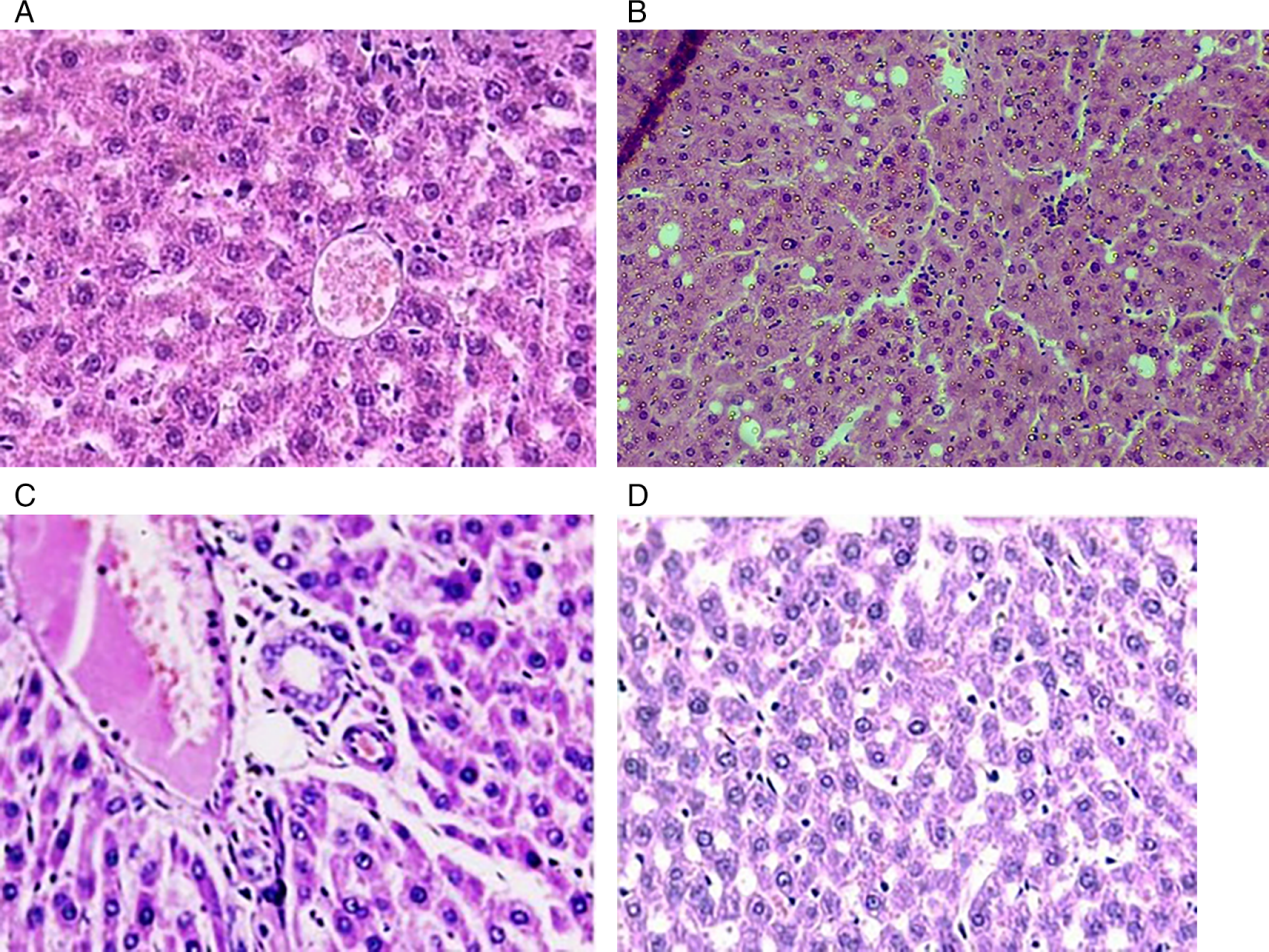
Cross sections (liver) for (a): control groups sections clarify central vein (CV), and normal hepatocyte (H). (b): Phenytoin group sections reveal necrosis (N) with biliary stasis (B.S), and focal degenerations (F.D), alongside inflammatory cells infilteration. (c): The (Phenytoin + N-acetylcysteine) group exhibits a reduction in necrosis, degeneration, and decrease in number of inflammatory cells. (d): N-acetylcysteine group show no remarkable pathology. H & E 40X.

In this experimental setting, the liver sections obtained from the control group displayed normal histological characteristics that were distinguished by a central vein encircled by hepatocytes arranged in a radial fashion. In contrast, liver samples from the phenytoin-treated group showed prominent features, such as substantial congestion, hepatocyte degeneration, biliary stasis, vacuolated cytoplasm, localized necrosis, and inflammatory cell infiltration (neutrophils, lymphocytes, and eosinophils). The phenytoin and N-acetylcysteine groups showed considerable reductions in inflammatory cells, necrosis, and degeneration in the liver. Moreover, normal hepatocyte plates and lobular architecture remained intact. Liver slices from the N-acetyl cysteine group showed no significant abnormal features ( Figure 2: a-d).

## Discussion

Phenytoin is one of the most widely used antiepileptic drugs in the management of various types of epilepsy. It is frequently utilized in outpatient settings and almost all emergency services worldwide. It is increasingly being utilized in therapeutic treatment of neuropathic pain, ^22^ hiccups, migraines, and wound healing; ^23^ however, its dosage should be adjusted to reduce the risk of unwanted effects, which could lead to its withdrawal even if it is beneficial. ^24^ Phenytoin is commonly included in the list of ten major causes of drug-induced acute liver failure. More than 10% of cases of acute phenytoin hepatitis accompanied by jaundice lead to fatality. If signs of jaundice or liver illness occur early during treatment, it is advisable to halt the use of phenytoin. ^23– 25^ Liver maintenance was evaluated by measuring ALT, AST, and ALP levels, which are enzymes that are mainly expressed at higher levels in the cytoplasm. During liver injury, these enzymes are released into the bloodstream in accordance with the severity of the liver damage. ^26^ Serum bilirubin level is an additional conventional marker of liver damage. The findings of our investigation indicate that phenytoin therapy resulted in varying degrees of biochemical changes in the liver enzymes of rats relative to other groups. Additionally, Phenytoin caused hepatic necrosis with focal degeneration and biliary stasis in rat livers, as shown by histopathological analysis. The obtained results were consistent with the findings of the investigated biochemical parameters. This study confirmed the hepatotoxic effects of phenytoin treatment. N-acetylcysteine (NAC) is a synthetic form of cysteine. It is widely recognized as an anti-inflammatory and antioxidant agent that provides hepatoprotection against liver injury caused by paracetamol. ^9^ Different mechanisms have been suggested to be involved in the pathogenesis of phenytoin-induced hepatotoxicity, including oxidative stress and depletion of antioxidants. ^3^ This provided a rationale for investigating the potential preventive benefits of NAC against PHT-induced hepatic damage caused by phenytoin. NAC mainly enhances the formation of glutathione and removes reactive oxygen species (ROS) generated during oxidative stress. ^9, 12, 16^ Moreover, various studies have examined the probable mechanisms that may explain the positive benefits of NAC in cases of nonparacetamol overdose. Previous studies have demonstrated that NAC stimulates guanylate cyclase activation, probably due to its anti-inflammatory, antioxidant, inotropic, and vasodilatory properties that enhance hepatic blood circulation and oxygen supply to essential organs. ^27^ NAC can mitigate endoplasmic reticulum tension and enhance mitochondrial function, both of which aid in liver protection against damage. ^28, 29^ The same results were observed in cell cultures treated with tuberculosis medicines, ^30^ which further enhanced the potential positive effects of this agent in protecting the liver. The current investigation found that concomitant administration of NAC and phenytoin for 45 days led to a considerable decrease in phenytoin-induced hepatotoxicity, as indicated by the reduction in TSB, ALT, and AST levels. Moreover, NAC restored normal liver histopathology. These results are in agreement with those of previous studies that have demonstrated the hepatoprotective effects of NAC against several hepatotoxic conditions. Eftikhari et al., ^31^ found that NAC effectively reduced the increased levels of ALT and AST in an animal model of liver injury associated with risperidone. Additionally, NAC has demonstrated a defense mechanism against liver damage caused by other hepatotoxic medicines and chemicals, including adriamycin, ^16^ azathioprine, ^32^ and dimethyl nitrosamine. ^33^ In summary, NAC at a daily dose of (200) revealed a valuable effect against phenytoin-induced toxicity, probably because NAC acts as a scavenger of free radicals and mediates the oxidative stress pathway.

## Conclusion

This study concluded that concomitant administration of NAC and phenytoin lowers the risk of phenytoin-induced hepatotoxicity. Moreover, this study confirmed that NAC is relatively safe when administered for a relatively prolonged period.

## Data Availability

**Figshare: Raw data for “Hepatoprotective potential of N-acetyl cysteine in rats with phenytoin induced liver injury”** https://doi.org/10.6084/m9.figshare.28598213.v3 ^34^

## Reporting guideline

The completed ARRIVE checklist has been deposited in Figshare.

Checklist title: Arrive checklist (Hepatoprotective potential of N-acetyl cysteine in rats with phenytoin induced liver injury).

DOI: https://doi.org/10.6084/m9.figshare.29185418.v2 ^35^

License: CC0

## References

[1] Ahmed SN Siddiqi ZA : Antiepileptic drugs and liver disease. *Seizure.* 2006;15(3):156–164. 10.1016/j.seizure.2005.12.009 16442314

[2] Smythe MA Umstead GS : Phenytoin hepatotoxicity: a review of the literature. *DICP.* 1989;23(1):13–18. 10.1177/106002808902300102 2655293

[3] Saraswathy GR Maheswari E Santhrani T : Reversal of phenytoin induced hepatotoxicity by alpha lipoic acid in rats. *Afr. J. Pharm. Pharmacol.* 2015;9(7):198–204.

[4] Björnsson E : Hepatotoxicity associated with antiepileptic drugs. *Acta Neurol. Scand.* 2008 Nov 1;118(5):281–290. 10.1111/j.1600-0404.2008.01009.x 18341684

[5] Ramezani V Tavakoli F Emami A : Comparison of the Hepatotoxicity of Carbamazepine, Sodium Valproate, Phenytoin, Lamotrigine, and Vigabatrin in a Rat Model. *Avicenna J. Pharm. Res.* 2022 Jun 30;3(1):17–22. 10.34172/ajpr.2022.1056 Reference Source

[6] Hadi NR Amber KI Aziz ND : Acute inflammatory response after novolimus drug eluting stent implantation in diabetic patients with coronary artery disease and the impact of coenzyme q10. *World Heart Journal.* 2018;10(1):33–44.

[7] El-Sayed A-AAA : Ameliorative and Anti-Inflammatory Properties of Thuja occidentalis in Phenytoin-Induced Hepatic and Renal Dysfunctions in Male Albino Rats. *Environment Asia.* 2023;16(1). 10.14456/ea.2023.14

[8] Samuni Y Goldstein S Dean OM : The chemistry and biological activities of N-acetylcysteine. *Biochim Biophys Acta - Gen Subj.* 2013;1830(8):4117–4129. 10.1016/j.bbagen.2013.04.016 23618697

[9] Millea PJ : N-acetylcysteine: multiple clinical applications. *Am. Fam. Physician.* 2009 Aug 1;80(3):265–269. 19621836

[10] Pei Y Liu H Yang Y : Biological activities and potential oral applications of N-acetylcysteine: progress and prospects. *Oxidative Med. Cell. Longev.* 2018;2018. 10.1155/2018/2835787 29849877 PMC5937417

[11] Cuzzocrea S Mazzon E Costantino G : Beneficial effects of n-acetylcysteine on ischemic brain injury. *Br. J. Pharmacol.* 2000;130(6):1219–1226. 10.1038/sj.bjp.0703421 10903958 PMC1572181

[12] De Andrade KQ Moura FA Dos Santos JM : Oxidative Stress and Inflammation in Hepatic Diseases: Therapeutic Possibilities of N-Acetylcysteine. *Int. J. Mol. Sci.* 2015;16:30269–30308. 10.3390/ijms161226225 26694382 PMC4691167

[13] Tenório MC Graciliano NG Moura FA : N-Acetylcysteine (NAC): Impacts on Human Health. *Antioxidants (Basel).* 2021 Jun 16;10(6):967. 10.3390/antiox10060967 34208683 PMC8234027

[14] Palacio JR Markert UR Martínez P : Anti-inflammatory properties of N-acetylcysteine on lipopolysaccharide-activated macrophages. *Inflamm. Res.* 2011;60:695–704. 10.1007/s00011-011-0323-8 21424515

[15] Maheswari E Saraswathy GRL Santhranii T : Hepatoprotective and antioxidant activity of N-acetyl cysteine in carbamazepine-administered rats. *Indian J. Pharm.* 2014;46(2):211–215. 10.4103/0253-7613.129321 24741196 PMC3987193

[16] Kaya S Yalcın T Tektemur A : N-Acetylcysteine may exert Hepatoprotective effect by regulating Meteorin-Like levels in Adriamycin-induced liver injury. *Cell Stress Chaperones.* 2023 Nov;28(6):849–859. 10.1007/s12192-023-01376-3 37670199 PMC10746670

[17] Sukumaran D Usharani P Paramjyothi GK : A study to evaluate the Hepatoprotective effect of N- acetylcysteine on anti-tuberculosis drug induced hepatotoxicity and quality of life. *Indian J. Tuberc.* 2023;70(3):303–310. 10.1016/j.ijtb.2022.05.012 37562904

[18] Ghafil F Hassan E Aziz N : Cardioprotective Potential of Celastrol in Sepsis-Induced Cardiotoxicity; Mouse Model of Endotoxemia. *Iran J. War Public Health.* 2023;15(4):361–367.

[19] Rashid AAM Ahmed IH Aziz HJ : Hepatoprotective Activity of Ezetimibe against Risperidone-Induced Liver Injury in Rats. *Med. J. Babylon.* April-June 2024;21(2):431–437. 10.4103/MJBL.MJBL_1510_23

[20] Percie du Sert N Hurst V Ahluwalia A : The ARRIVE Guidelines 2.0: updated guidelines for reporting animal research. 10.1111/bph.15193 PMC7393194 32662519

[21] Kadhim SH Mosa AU Ubaid MM : Hepatorenal protective activity of Artemisia against diclofenac toxicity in male rats. *Pan Afr. Med. J.* 2022 Dec 43;43:192. 10.11604/pamj.2022.43.192.36160 36942132 PMC10024554

[22] Kopsky DJ Keppel Hesselink JM : Single-Blind Placebo-Controlled Response Test with Phenytoin 10% Cream in Neuropathic Pain Patients. *Pharmaceuticals (Basel).* 2018 Nov 12;11(4):122. 10.3390/ph11040122 30424471 PMC6316219

[23] Keppel Hesselink JM : Phenytoin repositioned in wound healing: clinical experience spanning 60 years. *Drug Discov. Today.* 2018 Feb;23(2):402–408. 10.1016/j.drudis.2017.09.020 28993152

[24] Patocka J Wu Q Nepovimova E : Phenytoin – An anti-seizure drug: Overview of its chemistry, pharmacology and toxicology. *Food Chem. Toxicol.* 2020;142:111393. 10.1016/j.fct.2020.111393 32376339

[25] Chalasani N Bonkovsky HL Stine JG : Clinical characteristics of antiepileptic-induced liver injury in patients from the DILIN prospective study. *J. Hepatol.* 2022 Apr;76(4):832–840. 10.1016/j.jhep.2021.12.013 34953957 PMC8944173

[26] Alkiyumi SS Abdullah MA Alrashdi AS : Ipomoea aquatic extract shows protective action against thioacetamide-induced hepatotoxicity. *Molecules.* 2012;17(5):6146–6155. 10.3390/molecules17056146 22617138 PMC6269074

[27] Popescu M Bratu A Agapie M : The Use and Potential Benefits of N-Acetylcysteine in Non-Acetaminophen Acute Liver Failure: An Etiology-Based Review. *Biomedicine.* 2024;12(3):676. 10.3390/biomedicines12030676 38540289 PMC10967777

[28] Kazaz IO Demir S Yulug E : N-acetylcysteine protects testicular tissue against ischemia/reperfusion injury via inhibiting endoplasmic reticulum stress and apoptosis. *J. Pediatr. Urol.* 2019;15(3):253.e1–253.e8. 10.1016/j.jpurol.2019.02.005 30890312

[29] Zhou J Terluk MR Orchard PJ : N-Acetylcysteine Reverses the Mitochondrial Dysfunction Induced by Very Long-Chain Fatty Acids in Murine Oligodendrocyte Model of Adrenoleukodystrophy. *Biomedicines.* 2021 Dec 3;9(12):1826. 10.3390/biomedicines9121826 34944641 PMC8698433

[30] Singh M Sasi P Gupta VH : Protective effect of curcumin, silymarin and N-acetylcysteine on antitubercular drug-induced hepatotoxicity assessed in an in vitro model. *Hum. Exp. Toxicol.* 2012;31(8):788–797. 10.1177/0960327111433901 22318308

[31] Eftekhari A Ahmadian E Azarmi Y : In vitro/vivo studies towards mechanisms of Risperidone-induced oxidative stress and the protective role of coenzyme Q10 and N-acetylcysteine. *Toxicol. Mech. Methods.* 2016;26(7):520–528. 10.1080/15376516.2016.1204641 27387968

[32] Raza M Ahmad M Gado A : A comparison of Hepatoprotective activities of aminoguanidine and N-acetylcysteine in rat against the toxic damage induced by azathioprine. *Comp. Biochem. Physiol. Part C Toxicol. Pharmacol.* 2003;134(4):451–456. 10.1016/s1532-0456(03)00022-x 12727294

[33] Priya S Vijayalakshmi P Vivekanandan P : Influence of N-acetylcysteine against dimethylnitrosamine induced hepatotoxicity in rats. *Toxicol. Ind. Health.* 2011;27(10):914–922. 10.1177/0748233711399323 21558131

[34] Aziz N : Raw data.Dataset. *figshare.* 2025. 10.6084/m9.figshare.28598213.v3

[35] Aziz N : raw data.xlsxRaw data and ARRIVE checklistRaw dataRaw data.Dataset. *figshare.* 2025. 10.6084/m9.figshare.29185418.v2

