# Supplementary material for: Hepatoprotective potential of N-acetyl cysteine in rats with phenytoin induced liver injury
Source: F1000Res. 2025 Jun 17;14:593. [Version 1] doi: 10.12688/f1000research.163160.1 (PMC12464531; doi:10.12688/f1000research.163160.1)
Supplement: Peer review history

# Reviewer report: Reviewer response for version 1

**DOI:** 10.5256/f1000research.179464.r411832

**Published:** 2025-09-25

**Authors:** Sina Abdul Amir Kadhim

**Affiliations:** 1 Qadisiya University, Ad Diwaniyah, Iraq

The protective effect of N-acetyl cysteine (NAC) against phenytoin (PHT)-induced hepatotoxicity in rats was examined in this study. A common antiepileptic medication, phenytoin, is linked to liver damage via oxidative stress and inflammatory pathways. Four groups of twenty rats were created: phenytoin + NAC, NAC only, control, and phenytoin only. After 45 days, histopathological analysis and biochemical markers (ALT, AST, ALP, and total bilirubin) were conducted. Significant increases in liver enzymes and noticeable histological damage, such as necrosis, biliary stasis, and inflammatory infiltration, were observed in the phenytoin group. Rats given phenytoin + NAC, on the other hand, showed decreased inflammation, preserved liver architecture, and decreased levels of bilirubin, ALT, and AST. NAC by itself had no negative effects. The results validate that NAC is a safe and efficient hepatoprotective agent by significantly reducing phenytoin-induced liver injury through its antioxidant and anti-inflammatory properties.

 1. Condense sentences about the anti-inflammatory and antioxidant properties of NAC into a single, succinct statement.

  2. Table 1 is instructive, but the legend needs to provide a more thorough explanation of the labeling (a, b, c, and d for significance).

  3. Although there is some repetition of background information, the discussion effectively connects findings to literature.

 4. What led to the selection of 200 mg/kg/day of NAC? Did you take dose-response into account? 

 5. How certain can we be of your results' statistical power when each group consists of only five animals?

 6. In comparison to phenytoin alone, why did the NAC + phenytoin group's ALP not decrease significantly? 

 7. Were biomarkers of oxidative stress (like MDA and GSH) or inflammation (like TNF-α) measured?

Is the work clearly and accurately presented and does it cite the current literature?

Yes

If applicable, is the statistical analysis and its interpretation appropriate?

Yes

Are all the source data underlying the results available to ensure full reproducibility?

No source data required

Is the study design appropriate and is the work technically sound?

Yes

Are the conclusions drawn adequately supported by the results?

Yes

Are sufficient details of methods and analysis provided to allow replication by others?

Yes

Reviewer Expertise:

pharmacology

I confirm that I have read this submission and believe that I have an appropriate level of expertise to confirm that it is of an acceptable scientific standard.

# Reviewer report: Reviewer response for version 1

**DOI:** 10.5256/f1000research.179464.r395460

**Published:** 2025-07-29

**Authors:** Ahmed Sultan

**Affiliations:** 1 Professor, University of Al-Qadisiyah, College of pharmacy, Al Diwaniyah, Iraq

Title: Hepatoprotective Potential of N-Acetyl Cysteine in Rats with Phenytoin-Induced Liver Injury

 Abstract

 The abstract was written well but I think that the aim is best modified as (This study aimed to evaluate the protective effect of N-acetylcysteine (NAC) against PHT-induced hepatotoxicity in rats).

 Introduction

 The introduction was good but required short paragraphs about the number of studies that were carried out that investigated the potential hepatoprotective effects of NAC against various hepatotoxic substances.

 I suggest a paragraph included more detail about N-acetyl cysteine in introduction section

 Methods

 1.why you give first group normal saline?

 2. In the paragraph (The animals were acclimated for seven days prior to the experiment, were in good health, and were free of specific pathogens), how did you prove that the animals were free of pathogens? I suggest to remove this sentence.

 3. Please put a reference for the paragraph (The animals were maintained for 24 h following their last dose and then anesthetized with xylazine and ketamine (75 mg/kg). Also, the anesthetic dose of xylazine not mentioned.

 4. How were the animals sacrificed?

 5. This paragraph also needs reference (Biochemical markers, including enzymes that measure total serum bilirubin TSB, ALT, AST, and alkaline phosphatase activity, were assessed via the colorimetric method, utilizing cell biolabs assay kits in compliance with the manufacturer’s

 protocols).

 6. Histopathological analysis section also without reference.

 7. In Statistical analysis section, the SPSS version was not mentioned.

 4. Results

 1. In the introduction section, you discussed the proposed mechanisms behind NAC's hepatic protective effects, such as its antioxidant and anti-inflammatory properties; therefore, it would be beneficial to measure an inflammatory marker, such as the cellular expression of NFKB, along with antioxidant biomarkers in your study groups to confirm your findings.

 2. In the section on results of histopathology and the related figure 2, it is best to indicate the histopathological scoring features, like prominent features such as substantial congestion, hepatocyte degeneration, biliary stasis, vacuolated cytoplasm, localized necrosis, and inflammatory cell infiltration (neutrophils, lymphocytes, and eosinophils), with arrows to be clearly understood.

 5. Discussion

 The discussion section was good. It provided valuable insights and highlighted the key findings of the study. Overall, it effectively connected the research to the broader context of the field.

 6. Conclusion

 Conclusion It's best to mention a recommended note that further studies in humans are required. This will help to validate the findings and ensure their applicability to diverse populations. Additionally, exploring the underlying mechanisms could provide deeper insights into the observed effects.

 References

 The references were written well. They provided clear insights and supported the arguments effectively. Overall, the attention to detail in the citations enhanced the credibility of the work.

Is the work clearly and accurately presented and does it cite the current literature?

Yes

If applicable, is the statistical analysis and its interpretation appropriate?

I cannot comment. A qualified statistician is required.

Are all the source data underlying the results available to ensure full reproducibility?

Yes

Is the study design appropriate and is the work technically sound?

Yes

Are the conclusions drawn adequately supported by the results?

Yes

Are sufficient details of methods and analysis provided to allow replication by others?

Partly

Reviewer Expertise:

pharmacology and therapeutics

I confirm that I have read this submission and believe that I have an appropriate level of expertise to confirm that it is of an acceptable scientific standard.
